# Differentiating social preference and social anxiety phenotypes in fragile X syndrome using an eye gaze analysis: a pilot study

**DOI:** 10.1186/s11689-019-9262-4

**Published:** 2019-01-21

**Authors:** Michael P. Hong, Eleanor M. Eckert, Ernest V. Pedapati, Rebecca C. Shaffer, Kelli C. Dominick, Logan K. Wink, John A. Sweeney, Craig A. Erickson

**Affiliations:** 1 Cincinnati Children’s Hospital Medical Center, 3333 Burnet Ave, Cincinnati, OH 452292 USA; 2 University of Cincinnati, College of Medicine, 3230 Eden Ave, Cincinnati, OH 45267 USA

**Keywords:** Fragile X syndrome, Eye tracking, Social anxiety, Gaze aversion, Social interest, Autism

## Abstract

**Background:**

Fragile X syndrome (FXS) is the leading inherited cause of autism spectrum disorder, but there remains debate regarding the clinical presentation of social deficits in FXS. The aim of this study was to compare individuals with FXS to typically developing controls (TDC) and individuals with idiopathic autism spectrum disorder (ASD) across two social eye tracking paradigms.

**Methods:**

Individuals with FXS and age- and gender-matched TDC and individuals with idiopathic ASD completed emotional face and social preference eye tracking tasks to evaluate gaze aversion and social interest, respectively. Participants completed a battery of cognitive testing and caregiver-reported measures for neurobehavioral characterization.

**Results:**

Individuals with FXS exhibited reduced eye and increased mouth gaze to emotional faces compared to TDC. Gaze aversive findings were found to correlate with measures of anxiety, social communication deficits, and behavioral problems. In the social interest task, while individuals with idiopathic ASD showed significantly less social preference, individuals with FXS displayed social preference similar to TDC.

**Conclusions:**

These findings suggest fragile X syndrome social deficits center on social anxiety without the prominent reduction in social interest associated with autism spectrum disorder. Specifically designed eye tracking techniques clarify the nature of social deficits in fragile X syndrome and may have applications to improve phenotyping and evaluate interventions targeting social functioning impairments.

## Background

Fragile X syndrome (FXS) is the leading inherited cause of neurodevelopmental disability and is characterized by a CGG trinucleotide repeat expansion in the promoter region of the fragile X mental retardation 1 gene (FMR1) on the long arm of the X chromosome. Hypermethylation of the trinucleotide repeat expansion (≥ 200 CGG repeats is termed “full mutation” and causes FXS) leads to silencing of the FMR1 gene and marked reduction or loss of expression of the fragile X mental retardation protein (FMRP). Loss of FMRP causes a dysregulation of mRNA translation resulting in increased length and density of dendritic protrusions, decreased neural plasticity, and abnormal synaptic function [1, 2]. The FXS phenotype is associated with both cognitive impairment and behavioral abnormalities including sensory processing defects, inattention, and hyperactivity [3, 4]. In males, some degree of neuropsychiatric symptoms is nearly universal, but there is a wider variability of phenotype and deficits in heterozygous females due to random X inactivation patterns.

Anxiety is one of the hallmarks of the FXS behavioral phenotype with reports of over 70% of males and 56–77% of females reporting anxiety symptoms and meeting criteria for an anxiety disorder, predominantly social anxiety and specific phobias [5, 6]. Individuals with FXS present with clinically observable signs of social anxiety such as the “fragile X handshake,” an initiation of a typical social interaction or momentary eye contact directly followed by gaze aversion [7]. Anxiety is a critical deficit and key treatment target with a significant group of individuals with FXS treated for anxiety with antidepressants [8, 9]. In addition to pervasive social anxiety, FXS is also the most commonly observed single-gene cause of autism spectrum disorder (ASD) accounting for approximately 2–5% of cases [10, 11]. Overlapping symptomology with autism, such as poor eye contact, hand flapping, and hand biting, has been shown to be present as early as 2–5 years old [12]. Studies have found approximately 10–30% of individuals with FXS meeting full criteria for ASD, which consists of social deficits, repetitive behaviors, restrictive interests, and sensory hypersensitivity, and 90% of male children having one or more ASD features [13–15].

Eye tracking has swiftly become a popular investigational tool in developmental disabilities due to its ability to assess a wide range of cognitive processes even in the most impaired individuals [16–18]. Studying eye movements during face or social context viewing has created an avenue to quantify and objectively study social processing through an examination of gaze patterns [19]. Studies of ASD have shown reduced gaze towards social stimuli and increased orientation towards non-social objects and movement [20–22]. Additionally, studies of eye gaze during the viewing of emotional faces have revealed atypical face scanning associated with hypoactivation of the fusiform gyrus and hyperactivation of the amygdala in ASD [23, 24]. Studies of eye tracking in FXS are more limited but have shown results generally consistent with findings observed in autism, including reduced gaze towards the eyes and increased pupillary response [25, 26] as well as abnormal autonomic regulation when viewing faces [27]. These findings have translated to more naturalistic scenarios as well with FXS participants looking less at the face, exhibiting sympathetic and parasympathetic dysregulation associated with gaze aversion, and holding gaze for shorter time periods than controls when socially interacting with a female experimenter [28, 29].

Despite similarities in gaze tracking studies of ASD and FXS, some key differences have been observed. First, while social eye tracking in autism has shown variability of deficits across individuals and studies [30], studies of FXS have more consistently found gaze aversive behavior and elevated autonomic response to social stimuli [26, 28]. Additionally, limited evidence suggests mechanistic differences in brain imaging studies. In a study of emotional face processing, hypoactivation of the fusiform gyrus was observed in FXS similar to ASD, but individuals with FXS also demonstrated increased activation of the left hippocampus, left superior temporal gyrus, right insula, and left postcentral gyrus not observed in ASD. Hyperactivation of these other brain regions suggests the neural networks underlying social deficits in FXS may differ from those associated with social challenges in autism [31, 32] and may reflect a general reduction in functional habituation related to neural hyperexcitability in FXS [33, 34]. Furthermore, studies of individuals with FXS and male fragile X premutation carriers have found reduced amygdala activation while viewing fearful faces accompanied by altered activity of numerous other brain regions key in social cognition [35, 36]. Amygdala activation deficits were found to be related to both social functioning deficits as well as abnormal FMR1 gene expression and levels of FMRP [37]. Some have hypothesized an inability to recruit higher-level social processing regions during the memory encoding process to be a driving factor in FXS-specific social anxiety, but further research into these complex mechanisms is needed [38].

The aim of the current study was to quantify and dissociate social interest from social anxiety in FXS and ASD. In order to assess these features, we implemented two separate eye tracking paradigms: (1) a social preference task developed to assess social interest in ASD and other developmental disabilities across a wide range of intellectual functioning and verbal ability [16, 22] and (2) an emotional face gaze task applied in previous FXS eye tracking studies to examine gaze aversion [25, 26], which can result from social anxiety or reduced social interest. We hypothesized that individuals affected by FXS would demonstrate more social interest in the social preference task but have similar gaze aversion compared to individuals with ASD.

## Methods

### Subjects

Seventeen participants with a confirmed genetic diagnosis of fragile X syndrome (FXS) and 17 typically developing controls (TDC) with no significant medical, psychiatric, or neurological disease were recruited at Cincinnati Children’s Hospital through a broad cognitive and neurophysiological phenotyping study. All participants were evaluated by a child psychiatrist who specializes in FXS and ASD with inclusion of previous ADOS results when available. Of the 17 individuals with FXS enrolled, ADOS results were available for 8. Additionally, 17 individuals with a clinical diagnosis of idiopathic autism spectrum disorder (ASD) according to the ADOS and clinician evaluation were retrospectively selected based on the closest age- and gender-match from three other studies (by the same investigators) utilizing the same eye tracking paradigms. Participants were between 5 and 30 years of age, and all groups were age- and gender-matched. All participants or their guardians (as indicated) provided written informed consent for study participation, and the study protocol was approved by the local Institutional Review Board. Participants between 11 and 17 years of age and/or with a legal guardian provided written assent for study participation when possible.

### Measures

Cognitive and social functioning phenotyping was completed for FXS and TDC subjects. The Stanford-Binet fifth edition (SB5) routing administration consisting of nonverbal fluid reasoning and verbal knowledge was used to assess IQ [39]. Caregivers completed the following scales: (1) the Anxiety, Depression, and Mood Scale (ADAMS) to evaluate five behavioral dimensions: manic/hyperactive behavior, depressed mood, social avoidance, general anxiety, and compulsive behavior [40]; (2) the Aberrant Behavior Checklist - Community (ABC-C) to evaluate maladaptive behaviors, including irritability, lethargy, stereotypy, hyperactivity, inappropriate speech, and social avoidance [41]; and (3) the Social Communication Questionnaire (SCQ) to screen for behaviors related to autism spectrum disorder [42]. (4) The Vineland Adaptive Behavior Scale second edition (VABS-II) was completed for FXS subjects to assess adaptive functioning of FXS participants [43]. Additionally, the ABC-C and IQ testing were obtained for ASD subjects as a part of the other studies they were selected from. The ABC-C was scored using the reformulated FXS-specific factor structure [44].

### Apparatus, procedure, and stimuli

Eye tracking data was collected using a Tobii T300 infrared binocular eye tracker sampling gaze at a rate of 300 Hz. An integrated 17-in. flat-panel monitor running Tobii Studio was used for paradigm presentation (version 3.0, Tobii Technology, Sweden). Gaze data was analyzed using Tobii Studio (Tobii Pro, Stockholm, Sweden). Successful calibration using the Tobii Studio “5-point infant calibration” routine was required prior to starting the paradigm. Gaze testing was performed in a single session in a quiet room. Following calibration, participants were instructed to view the monitor throughout the task. Participants completed one social interest and one emotional face paradigm.

The social interest paradigm began with a 20-s fixation cross followed by three 20-s silent side-by-side social scene and geometric pattern videos for a total of 60 s used in previous studies of neurodevelopmental disorders [16, 22]. The side of the social scene video was switched after each 20-s segment. Subjects completed one of two variations of the social interest paradigm with the same social scenes and geometric patterns oppositely sided to account for side bias.

**Fig. 1:**
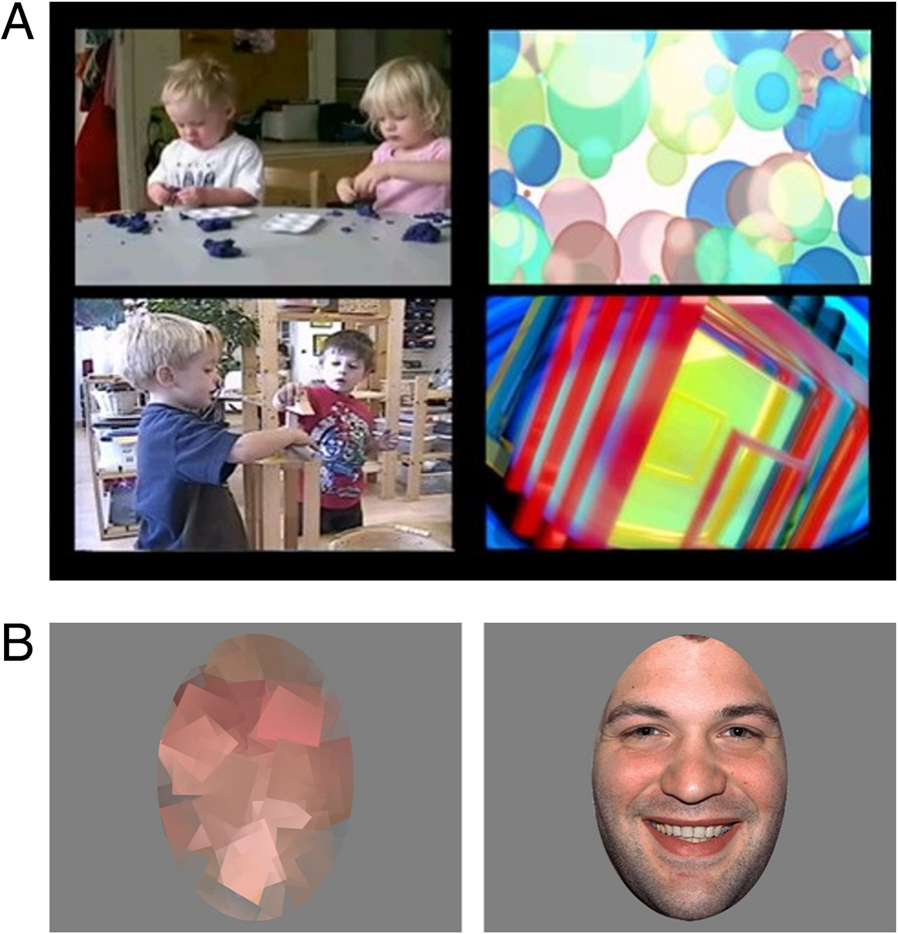
**a** Images of two examples of side-by-side social scene and geometric pattern videos displayed in the social preference paradigm and **b** an example of a scrambled face (left) and emotional face (right) from the emotional face paradigm

Following completion of the social interest paradigm, the emotional face paradigm was presented. The emotional face paradigm consisted of 12 emotional faces presented for 3 s per face. Happy, calm, and fear male and female faces previously used in studies of FXS eye tracking were utilized in this study [25, 26]. Each emotional face was preceded by a 1-s central fixation image of the Tobii Studio infant calibration and a 1-s scrambled version of face images (Fig. 1). Halfway through and following the completion of the 12 emotional faces, additional 20-s fixation crosses and 60-s side-by-side social scene and geometric pattern videos with audio from the social scenes were presented. Subjects completed one of two variations of the emotional face paradigm with different randomizations of the order of emotional faces. Depictions of the side-by-side social scenes and emotional faces are shown in Fig. 1.

### Analyses

#### Paradigm 1. Social scene preference ratio

Rectangular areas of interest (AOIs) were created around both the social scene and geometric pattern videos (Fig. 1). One TDC did not meet a priori cutoffs of greater than 10 s of the total viewing time and greater than 1 s viewing each of the social scene videos and geometric pattern videos during the 60-s side-by-side video segment and was excluded from the social scene preference ratio analysis. Social scene preference ratio (SSPR) was calculated by dividing the time spent viewing the social scene videos by the total time spent viewing the social scene or geometric pattern videos (Eq. 1). Only the silent side-by-side video paradigm was used in this analysis as audio from the social scene video was found to have a significant impact on selective preference towards the coinciding video.

1 $$ \mathrm{SSPR}=\frac{\mathrm{Viewing}\kern0.5em {\mathrm{Time}}_{\mathrm{Social}\kern0.5em \mathrm{Scenes}}}{\mathrm{Viewing}\kern0.5em {\mathrm{Time}}_{\mathrm{Total}}} $$

#### Paradigm 2. Emotional faces

Areas of interest for the eye, nose, mouth, and whole face used in prior FXS emotional face eye tracking studies were created [25, 26]. The “other” region was designated as any area within the face that was not within the eye, nose, or mouth region. Fixation counts (FC) to each region were calculated by averaging the number of fixations to the area of interest per face. Proportion looking time (PLT) was calculated by dividing the time spent on the area of interest over the total time spent viewing the face. Total emotional face viewing time was determined by the cumulative amount of time spent viewing the 12 emotional faces as a measure of overall gaze to emotional faces. Total scrambled face viewing time was determined by the cumulative amount of time spent viewing the 12 scrambled faces. Total scrambled face viewing time was utilized as a measure of overall ability to orient towards the paradigm and complete eye tracking unrelated to gaze aversion or social disinterest. One TDC, one FXS subject, and one ASD subject did not meet a priori cutoffs of greater than 4 of the 12 s of the paradigm’s total scrambled face viewing time and were excluded from the emotional face analysis. There was no minimum cutoff for total emotional face viewing time as to not exclude subjects based on gaze aversion-related behavior.

### Statistics

One-way analyses of variance (ANOVA) were conducted to assess group differences in clinical measures of cognition and social functioning. Post hoc comparisons were calculated for statistically significant results. Independent samples *t* tests were used for clinical measures obtained by only two groups.

To evaluate differences between FXS, ASD, and TDC groups on the social interest paradigm, a one-way analysis of covariance (ANCOVA) of SSPR with age as a covariate was performed. For statistically significant results, post hoc comparisons were calculated.

For the emotional face task, a one-way ANCOVA comparing total scrambled face viewing time with age as a covariate was computed to assess group differences in ability to eye track and orientation towards the screen. Post hoc comparisons were calculated for statistically significant results. A repeated measures analysis of covariance (RMANCOVA) for total emotional face viewing time with type of emotion as a within-subjects variable and group as a between-subjects variable was computed to evaluate differences in overall gaze to emotional faces. To evaluate differences in emotional face processing, a RMANCOVA was performed with type of emotion and face region as within-subjects variables and group as a between-subjects variable for both FC- and PLT-dependent variables. Total scrambled face viewing time and age were used as covariates for all RMANCOVAs to account for differences in ability to eye track and developmental stages respectively. Mauchly’s test of sphericity was used to assess variance of the differences between within-subjects conditions. For statistically significant results of the RMANCOVAs, post hoc comparisons were computed using the Bonferroni correction to account for the numerous comparisons in the models. Pearson product-moment correlations between clinical ratings and eye tracking measures with significant group differences as well as between measures of the two eye tracking paradigms were conducted for each group. The threshold for statistical significance in this study was set at a two-tailed *p* < 0.05.

## Results

### Subjects

Demographics, cognitive measures, and behavioral characterization are summarized in Table 1. Psychotropic medication information is summarized in Table 2. For various reasons, data was not available for three FXS participants on the SCQ; for two FXS participants on the VABS-II, ABC-C, and SB5; for one FXS participant on the ADAMS; for three ASD participants on the ABC-C; and for four ASD participants on IQ testing.

**Table 1:** Clinical characterization

	FXS	TDC	ASD
*N*	17	17	17
Age	16.6 ± 6.1	16.6 ± 5.8	16.5 ± 5.8
Gender (% male)	70.6	70.6	70.6
ASD (% clinically diagnosed)	17.6	0	100
IQ	58.6 ± 17.7^ac^	102.4 ± 12.3^b^	95.9 ± 29.8^b^
VABS-II Communication subscale	58.1 ± 18.2		
VABS-II Daily Living subscale	65.4 ± 10.8		
VABS-II Socialization subscale	64.7 ± 9.6		
VABS-II Adaptive Behavior Composite	61.2 ± 12.0		
SCQ	13.0 ± 7.7^a^	1.8 ± 1.9^b^	
ADAMS Manic/Hyperactivity subscale	7.0 ± 4.2^a^	0.8 ± 1.4^b^	
ADAMS Depressed Mood subscale	2.8 ± 3.5^a^	0.8 ± 1.4^b^	
ADAMS Social Anxiety subscale	7.2 ± 5.5^a^	1.1 ± 2.4^b^	
ADAMS Generalized Anxiety subscale	8.1 ± 5.7^a^	1.2 ± 1.8^b^	
ADAMS Compulsive Behavior subscale	2.1 ± 2.2^a^	0.2 ± 0.8^b^	
ABC-C Irritability	13.7 ± 14.1^a^	0.4 ± 1.0^bc^	12.4 ± 12.1^a^
ABC-C Lethargy	5.3 ± 5.4^a^	0.5 ± 1.7^bc^	8.8 ± 7.9^a^
ABC-C Stereotypy	2.7 ± 3.5^a^	0.0 ± 0.0^bc^	3.6 ± 3.6^a^
ABC-C Hyperactivity	8.5 ± 6.6^a^	0.5 ± 0.9^bc^	7.5 ± 5.2^a^
ABC-C Inappropriate Speech	4.5 ± 3.7^a^	0.1 ± 0.3^bc^	3.5 ± 2.8^a^
ABC-C Social Avoidance	3.3 ± 3.1^a^	0.5 ± 1.5^bc^	5.2 ± 4.4^a^

Descriptive statistics reported as means ± standard deviation

*FXS* fragile X syndrome, *TDC* typically developing control, *ASD* idiopathic autism spectrum disorder, *VABS-II* Vineland Adaptive Behavior Scales II, *SCQ* Social Communication Questionnaire, *ADAMS* Anxiety, Depression, and Mood Scale, *ABC-C* Aberrant Behavior Checklist - Community

^a^Statistically different from TDC (ANOVA followed by post hoc testing or independent samples *t* test)

^b^Statistically different from FXS (ANOVA followed by post hoc testing or independent samples *t* test)

^c^Statistically different from ASD (ANOVA followed by post hoc testing or independent samples *t* test)

**Table 2:** Psychotropic medications

	FXS	TDC	ASD
Antidepressants	9	0	7
Inattention/stimulants	7	0	7
Antipsychotics	3	0	5
Melatonin	4	0	6
Anticonvulsants	2	0	1
Acamprosate	2	0	0
Benzodiazepines	1	0	0
Triptans	0	0	1

Psychotropic medication use reported as number of subjects per group taking medications of each drug class

### Social interest

**Fig. 2:**
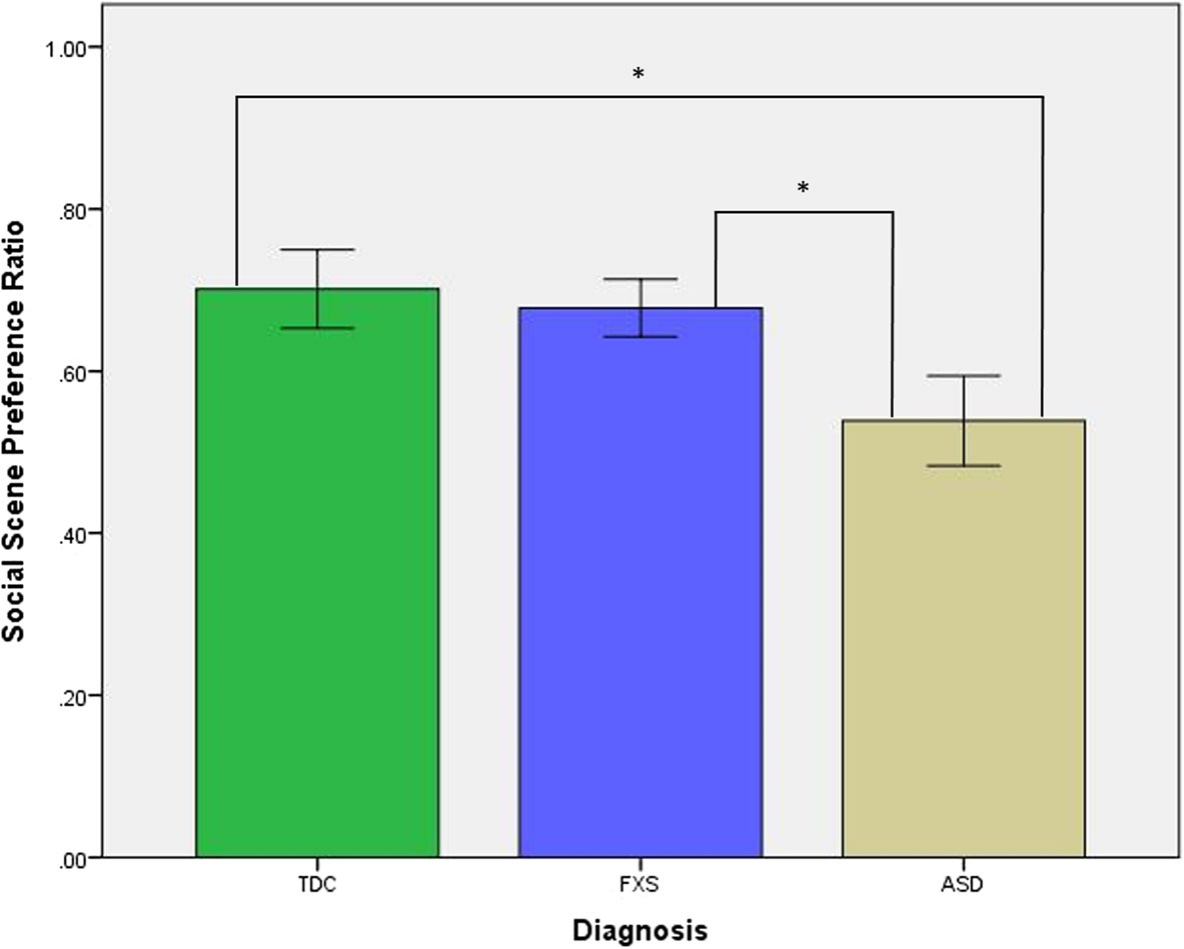
Mean social scene preference ratio (SSPR) ± standard errors of the mean by group (*p < 0.05)

Statistically significant group differences were observed on the social scene preference ratio (SSPR) [*F*(2, 46) = 3.40, *p* = 0.042]. Post hoc comparisons revealed the main effect was driven by a reduced SSPR in the ASD group compared to both FXS (*p* = 0.042) and TDC (*p* = 0.022) groups (Fig. 2). There was no significant difference in SSPR between the FXS and TDC groups. Additionally, there was no significant effect of age on SSPR.

### Emotional face processing

A statistically significant main effect of diagnosis on total scrambled face viewing time was found [*F*(2, 47) = 3.38, *p* = 0.043]. Post hoc comparisons revealed an effect of decreased total scrambled face viewing time in FXS compared to TDC (*p* = 0.017), suggesting individuals with FXS had more difficulty eye tracking and orienting towards the screen. There was no statistically significant effect of group, emotion, or group by emotion on the total emotional face viewing time.

For proportion looking time (PLT), Mauchly’s test for sphericity was violated for emotion (*χ*^2^(2) = 16.7, *p* < 0.05), region (*χ*^2^(5) = 21.1, *p* < 0.05), and emotion by region (*χ*^2^(20) = 84.3, *p* < 0.05). Thus, the Greenhouse-Geisser correction was used for tests of within-subjects effects. A statistically significant main effect of region [*F*(2.19, 94.11) = 5.73, *p* = 0.0035] and interaction effects of diagnosis by region [*F*(4.38, 188.34) = 2.45, *p* = 0.046] and emotion by region [*F*(3.48, 149.58) = 3.33, *p* = 0.016] were observed. There was no significant main effect of emotion by region by diagnosis. Further, there were no significant effects of age or total scrambled face viewing time on PLT. Post hoc comparisons of diagnosis by region revealed an effect of increased PLT to the mouth region in individuals with FXS compared to TDC (*p* = 0.020). No differences were found in the ASD and TDC or ASD and FXS comparisons.

**Fig. 3:**
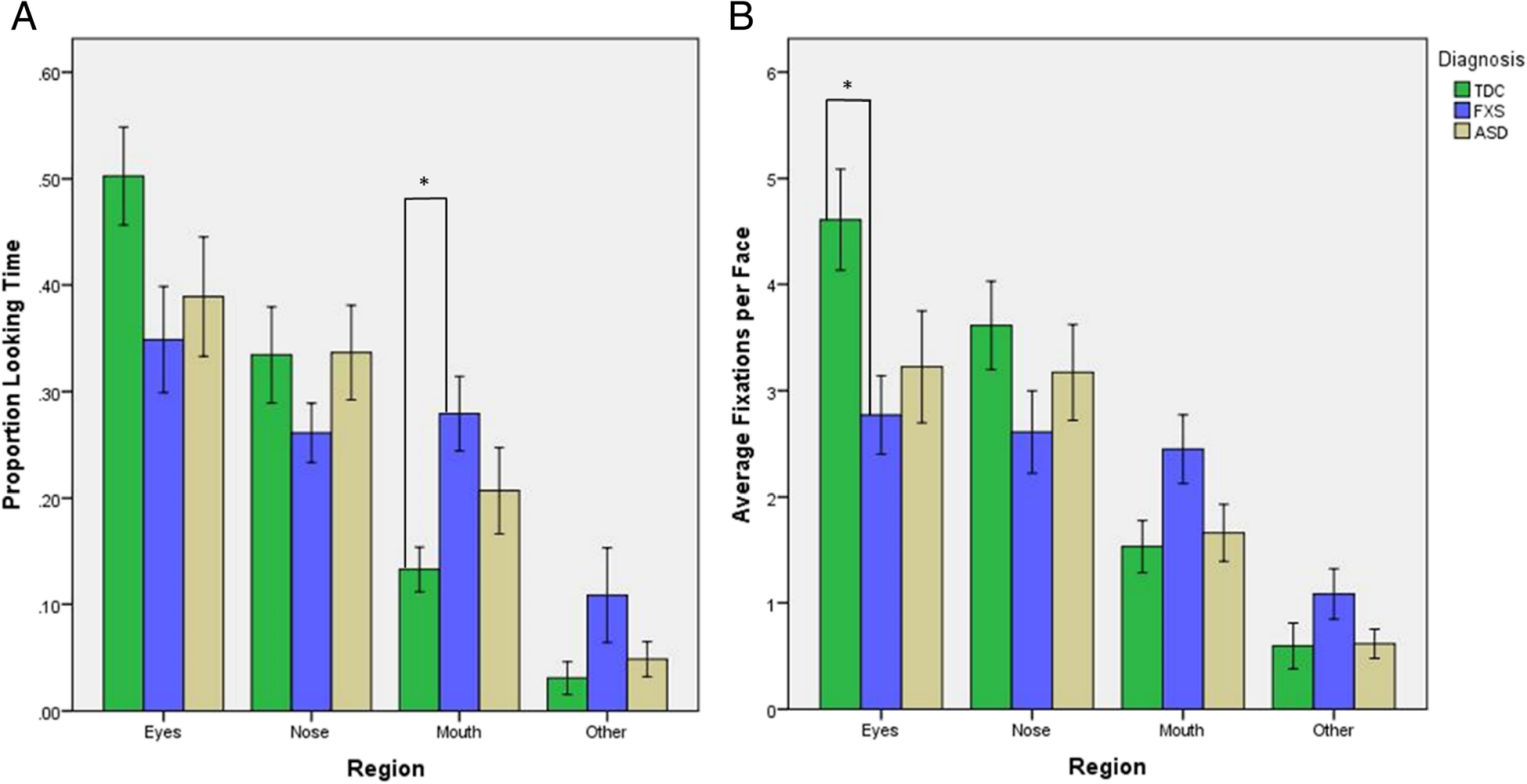
Mean **a** proportion looking time (PLT) per region by group and **b** average number of fixations to each region per face (FC) by group ± standard errors of the mean (*p < 0.05)

Mauchly’s test for sphericity was violated in the RMANCOVA of fixation count (FC) for region (*χ*^2^(5) = 29.0, *p* < 0.05) and emotion by region (*χ*^2^(20) = 73.8, *p* < 0.05) but not for emotion (*χ*^2^(2) = 5.99, *p* > 0.05). Greenhouse-Geisser corrections were used for tests of within-subjects effects involving region and emotion by region. A statistically significant main effect of region [*F*(2.06, 88.64) = 5.92, *p* = 0.0035] and interaction effects of diagnosis by region [*F*(4.12, 177.29) = 3.03, *p* = 0.021] and emotion by region [*F*(4.04, 173.56) = 4.66, *p* = 0.0013] were observed. There was no significant main effect of emotion by region by diagnosis, significant effects related to age, or significant effects related to total scrambled face viewing time. Post hoc comparisons of the diagnosis by region effect revealed a reduced fixation count to the eyes in individuals with FXS compared to TDC (*p* = 0.021). No differences were found between the ASD and TDC nor ASD and FXS comparisons (Fig. 3).

### Clinical correlates

**Fig. 4:**
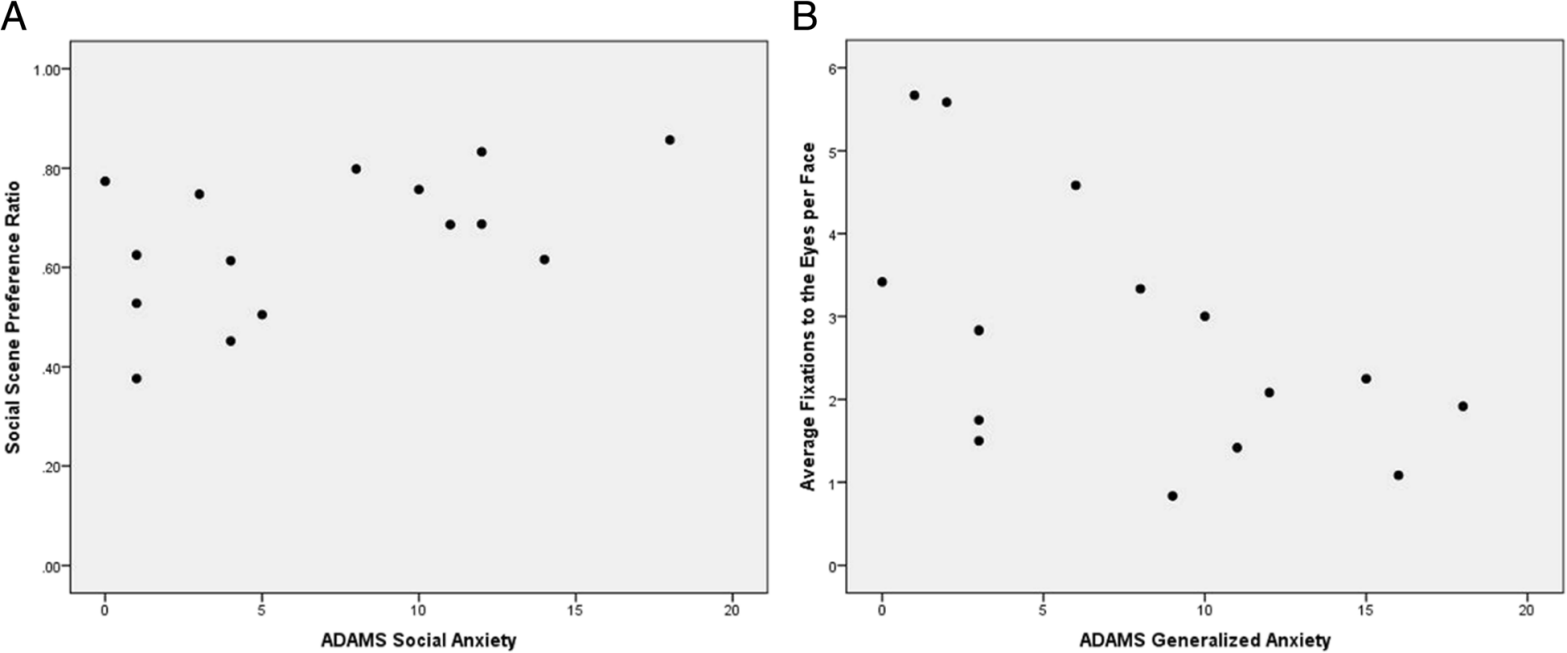
Pearson product-moment correlations of **a** ADAMS Social Anxiety with social scene preference ratio (SSPR) and **b** ADAMS Generalized Anxiety with average fixations to the eyes per face (FC to the eyes) in individuals with FXS

For FXS subjects, Pearson product-moment correlations of SSPR with clinical measures showed a positive correlation with the ADAMS Social Anxiety subscale (Fig. 4) suggesting more socially anxious individuals with FXS exhibited increased social preference [*r*(13) = 0.56, *p* = 0.026]. No correlations were observed between SSPR and age, abbreviated IQ, VABS-II, ABC-C, or SCQ scores.

Correlations of significant emotional face measures in FXS subjects revealed a negative correlation between FC to the eye region and the ADAMS Generalized Anxiety subscale [*r*(13) = − 0.55, *p* = 0.032] (Fig. 4) as well as the ADAMS Manic/Hyperactivity subscale [*r*(13) = − 0.59, *p* = 0.022], ABC-C Social Avoidance subscale [*r*(12) = − 0.59, *p* = 0.028], and SCQ [*r*(11) = − 0.55, *p* = 0.050]. Total emotional face viewing time was found to negatively correlate with the ABC-C Hyperactivity subscale [*r*(12) = − 0.53, *p* = 0.050]. No correlations were observed between significant emotional face eye tracking measures and age, VABS-II, IQ scores, or total scrambled face viewing time.

No correlation was observed between SSPR and emotional face eye tracking measures. In individuals with ASD, age was found to positively correlate with total emotional face viewing time [*r*(14) = 0.50, *p* = 0.047] and total scrambled face viewing time [*r*(14) = 0.59, *p* = 0.017]. In TDC, age was also found to positively correlate with total emotional face viewing time [*r*(14) = 0.69 *p* = 0.0029] and total scrambled face viewing time [*r*(14) = 0.61 *p* = 0.013]. These correlations suggest younger ASD and TDC participants had more difficulty eye tracking and orienting towards the screen. No other correlations between significant eye tracking and clinical or demographic measures were found in the TDC or ASD groups.

## Discussion

### The goal

The goal of the present study was to use eye gaze analysis to assess problems of social disinterest and social anxiety that can both occur and contribute to social difficulties in patients with fragile X syndrome (FXS) and idiopathic autism spectrum disorder (ASD). Using social preference and emotional face gaze analysis to study social interest and social anxiety symptomology has been done previously in studies of neurodevelopmental disorders for this purpose [16, 22, 26]. In this study, we observed that individuals with FXS demonstrated (1) an abnormal gaze pattern in the emotional face task suggestive of increased gaze aversion relative to typically developing individuals and (2) performance on the social preference test that not only suggested more social interest than in ASD, but that was not significantly different from TDC. These results have implications for a more in depth understanding of the social problems in FXS. Unlike ASD, which is more linked to reduced social interest than an anxiety-related response [45], individuals with FXS did not show reduced social interest but did exhibit increased gaze aversion. This suggests that while a high percentage of patients with FXS meet criteria for ASD, the underlying etiology of social symptoms in FXS patients may be fundamentally different from that of idiopathic ASD. Though the sample in the present study is small and replication is required, especially with validation in a larger group of individuals with comorbid FXS and ASD as well as an intellectually- and developmentally-matched control group, these paradigms may have significant implications for differential diagnosis of anxiety and social processing disorders as well as quantifying FXS-specific impairments in targeted behavioral and pharmacological therapeutic interventions for FXS patients.

First, emotional face processing findings of the reduced eye and increased mouth gaze coincide with prior studies of emotional faces in FXS [25, 26] and support our hypothesis of a pervasive social anxiety and gaze aversive phenotype. We also identified that fewer fixations to the eyes were associated with caregiver ratings of anxiety and social communication impairment in individuals with FXS. From a clinical perspective, social anxiety, including manifestations such as social withdrawal and gaze aversion, is a distressing and common symptom in individuals with FXS [5].

The novel finding of this study was the observation of gaze aversion but without the reduced social interest phenotypes of ASD in the FXS patients. Previous eye gaze studies of side-by-side social scenes and geometric patterns in ASD and other developmental disabilities have revealed distinct abnormalities suggesting reduced social interest and salience [16, 22, 46]. Considering this context, despite elevated social anxiety, individuals with FXS appear to have social interest and salience that remain relatively intact compared to ASD.

### Social anxiety in FXS

It has been widely reported that many FXS patients meet criteria for ASD and that FXS is the most common single-gene cause of ASD [10, 11]. However, it remains an open discussion in the field as to whether there are fundamental and mechanistic differences in the social phenotypes of FXS and idiopathic ASD. Individuals with FXS have a less variable developmental trajectory than ASD and often exhibit marked social anxiety as shown by gaze aversion and social avoidance as opposed to the social disinterest commonly observed in ASD [47, 48]. Individuals with FXS are often diagnosed with ASD [49, 50] but tend to more commonly meet diagnostic criteria for language/communication impairment and repetitive behaviors than social interaction criteria [48, 51, 52]. Due to high rates of intellectual disability in FXS, deficits in attention and working memory further contribute to communication and social difficulty in ways that behaviorally overlap with autism symptomology. While our findings indicate that social impairment in FXS may be more related to anxiety than social disinterest, additional studies evaluating autism symptoms in individuals with comorbid FXS and ASD are necessary. It also remains critical to continue developing techniques that can validly assess the multiple characteristics of social dysfunction involved with FXS-specific impairments, such as the gaze tracking approach of the present study.

Performance on the emotional face task was in some ways related to caregiver-reported measures of anxiety (Fig. 4). However, eye gaze abnormalities correlated with a range of other symptoms, including hyperactivity and social communication problems, and total emotional face viewing time correlated with hyperactivity as well indicating a broader clinical significance to atypical performance on this task. Conversely, while the social preference eye tracking paradigm successfully detected differences in social preference between FXS and ASD, it was not able to quantify social functioning difficulties related to social communication and interest across a spectrum of relevant parental ratings among individuals with FXS.

Interestingly, greater social preference was found to correlate with more severe social anxiety in the FXS group (Fig. 4). One likely explanation for this finding is that more socially anxious individuals are hypervigilant and attend more to social stimuli. Hypervigilance has been associated with anxiety disorders in the hypervigilance-avoidance hypothesis, which describes that anxious individuals will quickly focus on and then avoid threats [53, 54]. The exact mechanism regarding hypervigilant behavior in anxiety is not completely understood but may be specific to social anxiety [55]. Another possible explanation for this finding is that individuals with FXS with a higher level of social interest are more susceptible to social anxiety or more likely to have it generated by social interaction than their less socially cognizant counterparts.

### Technique

Methods of evaluating social behaviors and processing have made significant strides with the assistance of recent technological developments, such as gaze tracking. However, there is still much room for improvement in delineating specific social deficits using this approach. A critical future aim in the FXS research field is to develop symptom-specific evaluations that can characterize individuals on a continuum for use in phenotyping and intervention studies [7]. Using eye gaze studies to discriminate social anxiety and social interest challenges in FXS patients may prove to be useful tools for evaluating treatment response in clinical trials or for selecting individuals believed more likely to respond to a particular treatment in trials or clinical practice.

A more in-depth understanding of how FXS-specific symptoms impact social cognition and behavior may be utilized successfully in both psychological and pharmaceutical interventions in FXS treatment. These findings of typical social interest despite pervasive gaze aversive behavior suggest that therapies focused specifically on social anxiety may provide an effective route to improving social interactions versus more typical ASD social skill interventions focused on a lack of skill and motivation being the underlying causes of social impairments. For example, shaping procedures utilizing percentile schedules and pharmaceutical interventions using intranasal oxytocin have shown success in increasing eye contact in some individuals with FXS [56, 57]. While there have been few behavioral intervention studies in FXS to date [15], eye gaze assessment related to social and emotional circumstances may aid in social symptomology detection and impairment quantification for behavioral and pharmaceutical treatment research in FXS.

### Limitations

The results of this study must be considered in the context of its limitations. First, both the TDC and ASD groups had a significantly higher IQ than the FXS group. While no significant correlations with IQ were observed in this study, group differences in IQ must be considered for these social gaze findings. It is critical in future studies to evaluate an IQ-matched control group to determine the effect of intellectual and developmental functioning on the mechanisms underlying social gaze behavior in FXS. Further, due to sample size limitations, there was a limited ability to evaluate the impact of demographic and cognitive factors, such as age, gender, and IQ, as well as potential differences in FXS phenotypic subgroups on social gaze behavior. Specifically, a larger, adequately powered study comparing individuals with FXS with and without comorbid ASD may determine how presence of ASD behaviors impact social gaze behavior in FXS. In addition, while there were no differences found in social interest between FXS and TDC in this study, a larger sample size may elucidate if there are more subtle social interest problems in FXS. Notably, in the emotional face paradigm, there was no effect of type of emotion on group differences in gaze behaviors. A longer paradigm with more trials for each emotion may help differentiate emotion-related differences in face processing for individuals with FXS. In addition, in the present study, subjects passively viewed social scenes or photographs of faces, but interactive social tasks may more accurately evaluate social functioning. Finally, as discussed previously, continuing to develop social gaze measures that more specifically target distinct social behaviors is a goal for future studies which could be achieved with continued paradigm development and new analysis techniques.

## Conclusions

In the present study, individuals with FXS were found to show typical social preference and elevated gaze aversion utilizing quantitative social gaze tracking paradigms. Social interest and gaze aversion eye tracking paradigms provide novel, valid strategies for elucidating the mechanistic differences between FXS and idiopathic ASD with regard to causes for their social symptomology.

## Abbreviations

ABC-C: Aberrant Behavior Checklist - Community
ADAMS: Anxiety, Depression, and Mood Scale
ANCOVA: Analysis of covariance
AOIs: Areas of interest
ASD: Autism spectrum disorder
FC: Fixation count
FMR1: Fragile X mental retardation 1 gene
FMRP: Fragile X mental retardation protein
FXS: Fragile X syndrome
PLT: Proportion looking time
RMANCOVA: Repeated measures analysis of covariance
SB5: Stanford-Binet fifth edition
SCQ: Social Communication Questionnaire
SSPR: Social scene preference ratio
TDC: Typically developing control
VABS-II: Vineland Adaptive Behavior Scale second edition
